# Screening and Identification of the Metabolites in Rat Plasma and Urine after Oral Administration of *Areca catechu* L. Nut Extract by Ultra-High-Pressure Liquid Chromatography Coupled with Linear Ion Trap–Orbitrap Tandem Mass Spectrometry

**DOI:** 10.3390/molecules22061026

**Published:** 2017-06-21

**Authors:** Lulu Li, Zhiqiang Luo, Yang Liu, Hao Wang, Aoxue Liu, Guohua Yu, Mengwei Li, Ruirui Yang, Xinjing Chen, Jialian Zhu, Baosheng Zhao

**Affiliations:** 1 School of Chinese Materia Medica, Beijing University of Chinese Medicine, No. 6, Zhonghuan South Road, Wangjing, Chaoyang District, Beijing 100102, China; lulu130901@126.com (L.L.); lzq4y3r@126.com (Z.L.); adopus@126.com (H.W.); axljiayoula@163.com (A.L.); sufei_sophie@163.com (G.Y.); 20160931848@bucm.edu.cn (M.L.); yangrr5021@126.com (R.Y.); chxj9208@sina.com (X.C.); zhujialian2013@126.com (J.Z.); 2 Research Institute of Chinese Medicine, Beijing University of Chinese Medicine, No. 11, North Third Ring Road, Chaoyang District, Beijing 100029, China

**Keywords:** *Areca catechu* L., major compounds, metabolites, UHPLC-LTQ-Orbitrap

## Abstract

*Areca catechu* L. nut, a well-known toxic traditional herbal medicine, has been widely used to treat various diseases in China and many other Asian countries for centuries. However, to date the in vivo absorption and metabolism of its multiple bioactive or toxic components still remain unclear. In this study, liquid chromatography coupled with tandem mass spectrometry was used to analyze the major components and their metabolites in rat plasma and urine after oral administration of *Areca catechu* L. nut extract (ACNE). A total of 12 compounds, including 6 alkaloids, 3 tannins and 3 amino acids, were confirmed or tentatively identified from ACNE. In vivo, 40 constituents, including 8 prototypes and 32 metabolites were identified in rat plasma and urine samples. In summary, this study showed an insight into the metabolism of ACNE in vivo, which may provide helpful chemical information for better understanding of the toxicological and pharmacological profiles of ACNE.

## 1. Introduction

Traditional Chinese medicine (TCM) has played an important role in preventing or treating a variety of complicated diseases over thousands of years [1,2,3]. It is extensively accepted that the therapeutic or toxic effect of TCM results from the prototype components and/or their metabolites [4,5]. However, profiling the absorbed components and metabolites of an herbal medicine in vivo is always a great challenge [6]. Metabolites exist in a variety of forms and are usually present at trace levels; therefore, the signals from the metabolites are often masked by background noise from endogenous interference [7]. Owing to its high selectivity and sensitivity, liquid chromatography coupled with tandem mass spectrometry (LC–MS/MS) has been extensively used in drug metabolism studies. LC–MS/MS combined with sophisticated software tools can offer abundant structural information and accurate mass measurement for both precursor and product ions, which represents a powerful and reliable analytical technique to detect and identify unknown metabolites in complex matrixes [8,9,10,11,12].

*Areca catechu* L. one of most popular medicinal herbs, is widely distributed in the south of China. The nut of *Areca catechu* L. has been recorded in the pharmacopoeia of China for the treatment of parasitic diseases, dyspepsia, abdominal pain, etc. [13]. Modern pharmacological studies and clinical practice revealed that *Areca catechu* L. nut shows a variety of pharmacological functions, including wound healing [14], anti-migraine effect [15], anti-depressant effect [16], and hypoglycemic [17] and antioxidant effects [18]. As a toxic herbal medicine, *Areca catechu* L. nut also has some potential toxicities, such as decline of sperm count sand motility and induction of substantial abnormalities [19,20]. Although many reports regarding the pharmacological activities and potential toxicities have been published as described above, very little is known about its absorbed components and metabolites in vivo.

Hence, the overall objective of this research was to investigate in detail the in vivo absorption and metabolism of *Areca catechu* L. nut extract (ACNE). As we all know, plasma and urine are recognized as ideal biological matrices in drug absorption and metabolism studies [21,22]. In this work, a total of 26 components in the plasma and 31 components in the urine were characterized by ultra-high-pressure liquid chromatography coupled with linear ion trap-Orbitrap tandem mass spectrometry (UHPLC-LTQ-Orbitrap). From the results, we can know the probable metabolic pathways of chemical constituents in ACNE. To the best of our knowledge, this is the first study on screening the multiple absorbed and metabolic components of ACNE in vivo, which could provide a scientific basis for explaining the curative and toxicological mechanism of ACNE.

## 2. Results and Discussion

### 2.1. Identification of the Major Components in ACNE by UHPLC-LTQ-Orbitrap

**Figure 1:**
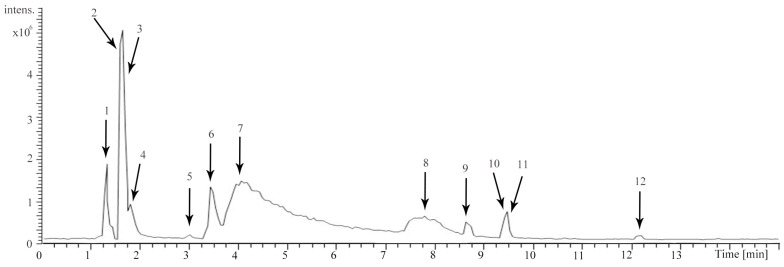
Extracted ion chromatogram of *Areca catechu* L. nut extract (ACNE) in positive ion mode.

**Figure 2:**
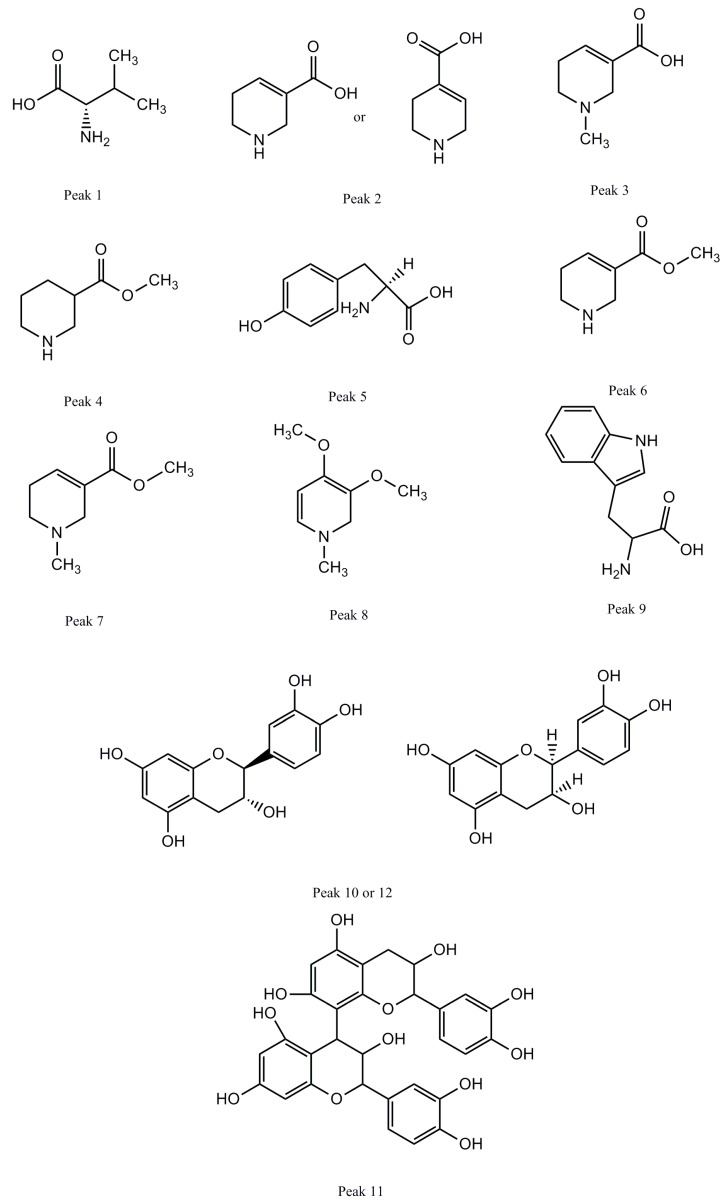
The structures of the 12 compounds.

A total of 12 compounds were separated and identified according to the retention time, obtained mass spectra, and by comparing with data from literature and standard sample. All of them were attributed to three types, including 6 alkaloids, 3 tannins and 3 amino acids. Detailed identified information is listed in Table 1. The detailed extracted ion chromatography of ACNE is shown in Figure 1 and Figure S1 in the Supplemental material. The structures of the 12 compounds are shown in Figure 2.

#### 2.1.1. Alkaloids

**Figure 3:**
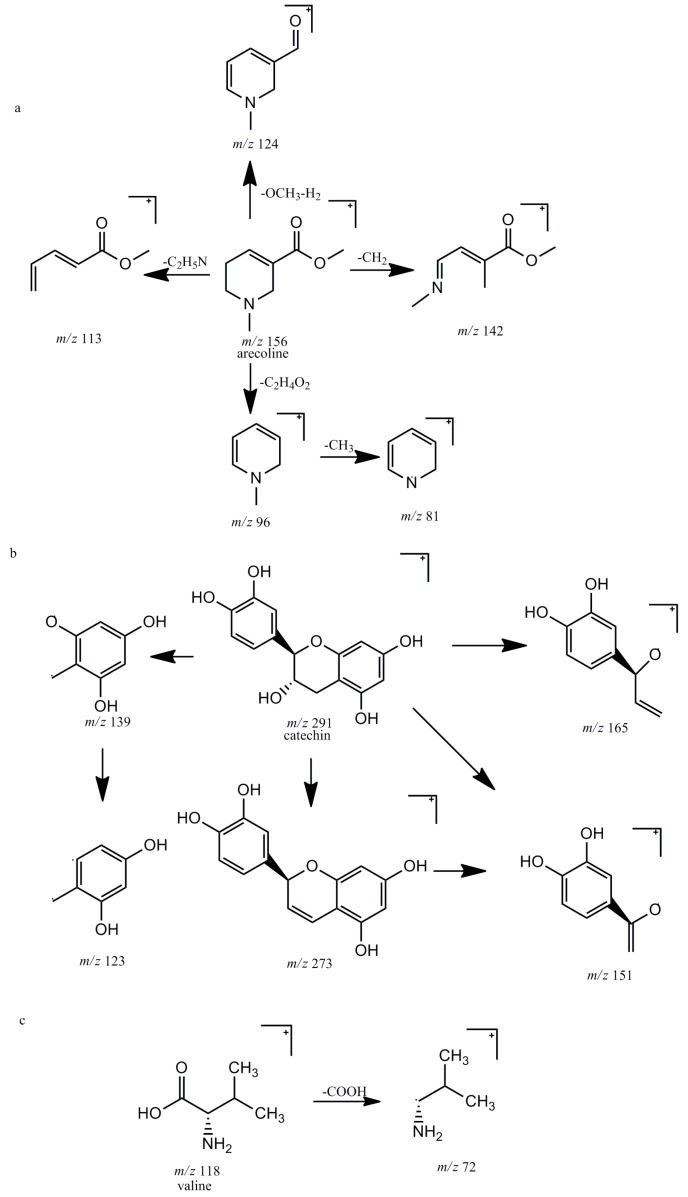
The proposed fragmentation pathways of arecoline (**a**); catechin (**b**) and valine (**c**) in positive ion mode.

Component **7** (4.09 min, −0.115 ppm) showed the accurate [M + H]^+^ ion at *m*/*z* 156.1018 and produced the characteristic ions at *m*/*z* 124.0757 [M + H-OCH_3_-H_2_]^+^, 113.0597 [M + H-C_2_H_5_N]^+^, 96.0807 [M + H-C_2_H_4_O_2_]^+^ and 81.0333 [M + H-C_2_H_4_O_2_-CH_3_]^+^. By comparing with the reference compound and the literature data [23], component **7** was identified as arecoline. The proposed fragmentation pathway of arecoline is shown in Figure 3a.

Component **8** (7.81 min, −0.255 ppm) displayed the molecular ion at *m*/*z* 156.1017. Moreover, it exhibited three major ions at *m*/*z* 128.1069 [M + H-2CH_3_]^+^, 113.0597 [M + H-3CH_3_]^+^ and 81.0333 [M + H-2OCH_3_-CH_3_]^+^. Accordingly [24], component **8** was tentatively identified as arecolidine.

Component **2** (1.70 min, −0.743 ppm) provided the [M + H]^+^ ion at *m*/*z* 128.0705, which led to two major product ions at *m*/*z* 110.0601 [M + H-H_2_O]^+^ and 81.0334 [M + H-COOH-H_2_]^+^. In accordance with the literature data [25], component **2** was tentatively confirmed as guvacine or its isomer, isoguvacine.

Component **3** (1.74 min, −0.085 ppm) exhibited the molecular ion at *m*/*z* 142.0862. It gave rise to four fragment ions at *m*/*z* 128.0745 [M + H-CH_2_]^+^, 124.0758 [M + H-H_2_O]^+^, 96.0807 [M + H-CH_2_O_2_]^+^ and 81.0333 [M + H-CH_2_O_2_-CH_3_]^+^. According to the literature data [26], component **3** was identified as arecaidine.

Component **6** (3.47 min, −0.115 ppm) also showed the molecular ion at *m*/*z* 142.0862 which was a 14-Da (CH_2_) loss of component **7**. Meanwhile, component **6** produced fragment ions at *m*/*z* 113.9638 [M + H-CH_3_N]^+^, 110.0602 [M + H-OCH_3_-H_2_]^+^ and 81.0334 [M + H-C_2_H_4_O_2_]^+^. By comparing with the reference compound and the literature data [25], component **6** was confirmed as guvacoline.

Component **4** (1.87 min, −0.869 ppm) yielded molecular ion at *m*/*z* 144.1018. Moreover, it showed major fragment ions at *m*/*z* 126.0915 [M + H-CH_3_-H_2_]^+^, 114.0913 [M + H-CH_3_N]^+^ and 84.0806 [M + H-C_2_H_4_O_2_]^+^. Thus, component **4** was tentatively identified as methyl piperidine-3-carboxylate.

#### 2.1.2. Tannins

Component **10** (9.48 min, −0.669 ppm) and component **12** (12.20 min, −0.978 ppm) showed the same [M + H]^+^ ion at *m*/*z* 291.0861, and both of them produced the same fragment ions at *m*/*z* 273.0762 [M + H-H_2_O]^+^, 165.0548 [M + H-C_6_H_6_O_3_]^+^, 151.0392 [M + H-C_7_H_7_O_3_]^+^, 139.0392 [M + H-C_8_H_7_O_3_]^+^ and 123.0443 [M + H-C_8_H_7_O_3_-OH]^+^. According to the literature data [27], they were tentatively designated as catechin (C) and epicatechin (EC), respectively. The proposed fragmentation pathway of catechin is shown in Figure 3b.

Compound **9** (8.68 min, −1.334 ppm) exhibited the [M + H]^+^ ion at *m*/*z* 579.1489, and gave rise to four major product ions at *m*/*z* 453.1197, 427.1039, 409.0937 and 291.0874. The fragment ion at *m*/*z* 409.0937 was generated through the loss of H_2_O from the ion at *m*/*z* 427.1039; the fragment ion at *m*/*z* 427.1039 was attributed to the retro Diels-Alder (RDA). The ion at *m*/*z* 291.0874 [M + H-C_15_H_13_O_6_]^+^ was a prototype losing an aggregate unit. According to the literature data [28], component **9** was tentatively characterized as procyanidin B2.

#### 2.1.3. Amino Acid

Component **1** (1.36 min, –1.568 ppm) displayed the molecular ion at *m*/*z* 118.0861 which led the major product ion at *m*/*z* 72.0806 [M + H-HCOOH]^+^. By comparing with the reference compound, component **1** was identified as valine. The proposed fragmentation pathway of valine is shown in Figure 3c.

Compound **5** (3.05 min, −0.822 ppm) exhibited [M + H]^+^ ion at *m*/*z* 182.0810 and the main fragment ions of it were at *m*/*z* 165.0548 and 136.0760. The former ion at *m*/*z* 165.0548 was produced from the neutral loss of NH_3_, and the latter ion at *m*/*z* 136.0760 was generated through the loss of HCOOH from the molecular ion. Hence, compound **5** was tentatively identified as tyrosine.

Compound **11** (9.53 min, −0.996 ppm) showed the [M + H]^+^ ion at *m*/*z* 205.0970 and it yielded major ions at *m*/*z* 188.0917 [M + H-NH_3_]^+^ and 159.9331 [M + H-HCOOH]^+^. Based on these data, component **11** was tentatively assigned as tryptophan.

### 2.2. Identification of the Major Metabolites in ACNE by UHPLC-LTQ-Qrbitrap

**Figure 4:**
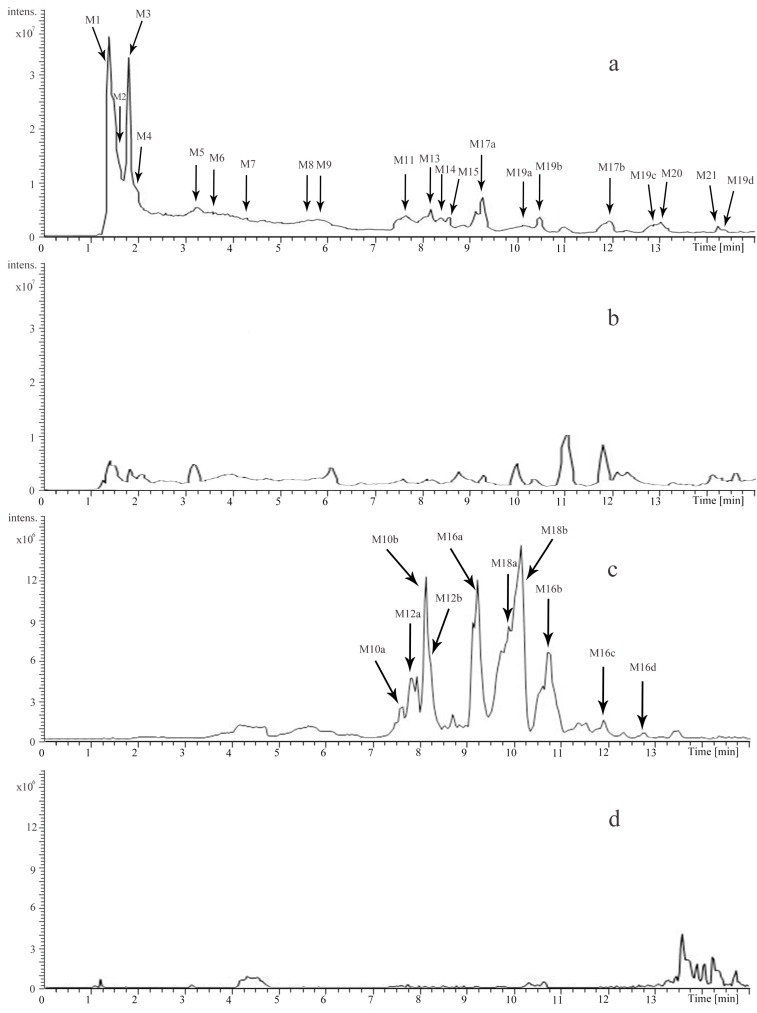
Extracted ion chromatograms of the urine sample in positive ion mode (**a**); the blank urine sample in positive ion mode (**b**); the urine sample in negative ion mode (**c**); the blank urine sample in negative ion mode (**d**).

**Figure 5:**
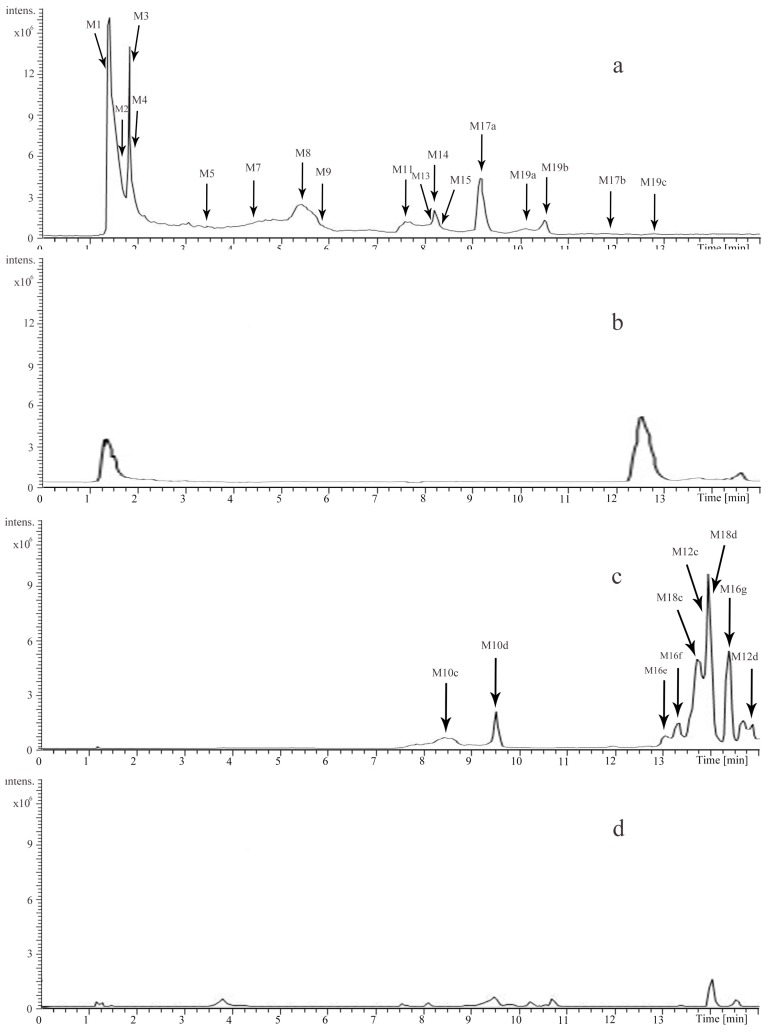
Extracted ion chromatograms of the plasma sample in positive ion mode (**a**); the blank plasma sample in positive ion mode (**b**); the plasma sample in negative ion mode (**c**); the blank plasma sample in negative ion mode (**d**).

**Figure 6:**
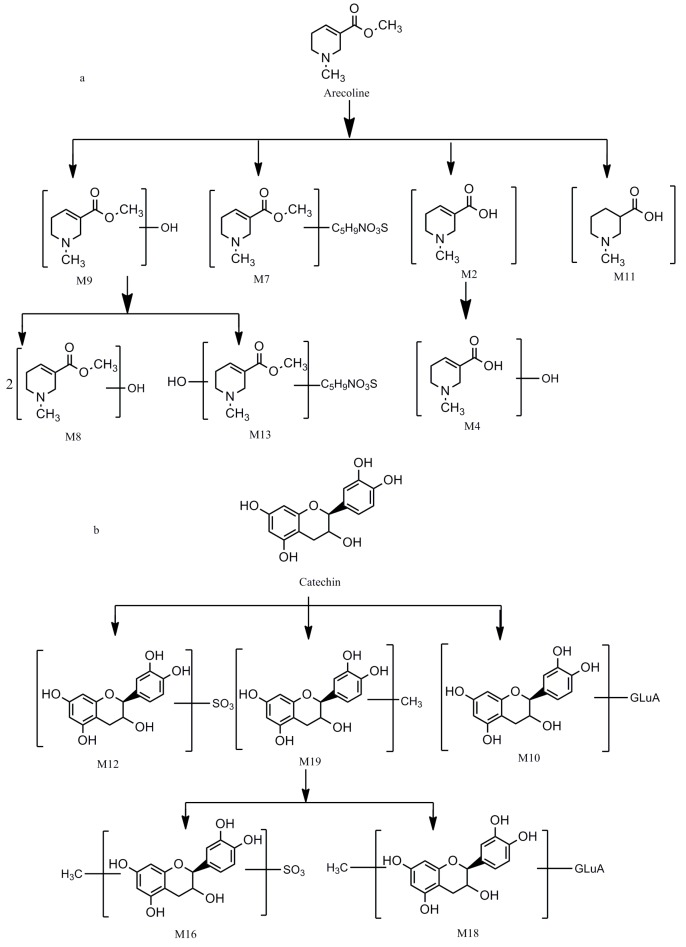
The proposed metabolic pathways of arecoline (**a**) and catechin (**b**).

The identification of the compounds in rat urine and plasma were also performed by UHPLC-LTQ-Qrbitrap. Altogether, 40 compounds, including 8 prototype and 32 metabolites were identified by comparing the retention time and the mass data (Table 2). Among them, 31 of them were from rat urine while 26 were from rat plasma. Detailed extracted ion chromatograms of the urine sample are shown in Figure 4, and the plasma sample are shown in Figure 5. In addition, the metabolic pathways of catechin (C) and arecoline are summarized in Figure 6.

#### 2.2.1. Prototypes (M1, M2, M3, M5, M6, M15, M17)

M1 (1.75 min, −0.352 ppm) showed the [M + H]^+^ ion at *m*/*z* 128.0706, and gave rise to two product ions at *m*/*z* 110.0601 and 81.0332, which were consistent with the product ions of component **2**. Therefore, M1 was identified as guvacine or isoguvacine.

M2 (1.76 min, −0.740 ppm) displayed the [M + H]^+^ ion at *m*/*z* 142.0862, and produced fragment ions at *m*/*z* 124.0759, 96.0809 and 81.0334 corresponding to component **3**. Thus, M2 was tentatively identified as arecaidine.

M3 (1.78 min, −0.661 ppm) showed the [M + H]^+^ ion at *m*/*z* 144.1018, and it produced the fragment ions at *m*/*z* 126.0915, 114.0915 and 84.0806 corresponding to component **4**. Based on these data, M3 was identified as methyl piperidine-3-carboxylate.

M5 (3.28 min, −0.388 ppm) displayed the same [M + H]^+^ ion at *m*/*z* 142.0862. In the MS/MS spectrum, M5 led three major fragment ions at *m*/*z* 113.0597, 110.0601 and 81.0333 which were consistent with component **6**. Therefore, M5 was tentatively identified as guvacoline.

M6 (3.58 min, −0.418 ppm) displayed the [M + H]^+^ ion at *m*/*z* 156.1018. In the MS/MS spectrum, M6 yielded ions at *m*/*z* 124.0758, 113.0598, 96.0807 and 81.0333, which were the diagnostic ions of component **7**. Thus, M6 was confirmed as arecoline.

M15 (8.60 min, −1.023 ppm) exhibited the [M + H]^+^ ion at *m*/*z* 579.1491, and led to four major product ions at *m*/*z* 453.1199, 427.1041, 409.0938 and 291.0875, which were the characteristic fragment ions of component **9**. Consequently, M15 was tentatively considered to be procyanidin B2.

M17a (9.31 min, 0.602 ppm) and M17b (11.99 min, 0.293 ppm) gave the same molecular ion at *m*/*z* 291, and in the MS/MS spectrum both of them displayed the fragment ions at *m*/*z* 273.0766, 165.0550, 151.0394, 139.0394 and 123.0444, corresponding to components **10** and **12**. Thus, M17a and M17b were confirmed as catechin (C) and epicatechin (EC), respectively.

#### 2.2.2. Methylated Metabolites (M19, M21)

M19a (10.16 min, 0.083 ppm), M19b (10.50 min, 0.280 ppm), M19c (12.94 min, −0.015 ppm) and M19d (14.35 min, −0.507 ppm) showed the same [M + H]^+^ ion at *m*/*z* 305, which was an increase of 14 Da (CH_2_) when compared with that of M17 (C/EC). Further MS^2^ spectra of them showed product ions at *m*/*z* 287.0921 [M + H-H_2_O]^+^ and 179.0707 [M + H-C_6_H_6_O_3_]^+^, indicating the methylation was asserted on the B ring. Based on these data, M19a-d were tentatively assessed as methylated C/EC.

M21 (14.27 min, −2.549 ppm) gave the [M + H]^+^ ion at *m*/*z* 321.0943, which was also an increase of 14 Da (CH_2_) when compared with that of epigallocatechin (EGC). In the MS/MS spectrum, M21 exhibited the fragment ions at *m*/*z* 303.1331 [M + H-H_2_O]^+^ and 289.0689 [M + H-H_2_O-CH_3_]^+^. In accordance with [29], M21 was identified as methylated EGC.

#### 2.2.3. Methylated and Sulfated Metabolites (M16)

M16a (9.20 min, 1.020 ppm), M16b (10.73 min, −0.259 ppm), M16c (11.73 min, −1.225 ppm), M16d (12.72 min, −0.729 ppm), M16e (13.01 min, 1.334 ppm), M16f (13.29 min, 0.289 ppm) and M16g (14.34 min, 2.378 ppm) displayed the same [M − H]^−^ ion at *m*/*z* 383, which was 80 Da (SO_3_) higher than that of M19, suggesting M16a-g were the sulfate conjugations of M19. Furthermore, the fragment ion of M16 at *m*/*z* 137.0249 [M − H-SO_3_-C_8_H_7_O_3_]^−^ indicated that the sulfation occurred on the A ring.

#### 2.2.4. Methylated and Glucuronidated Metabolites (M18)

M18a (9.86 min, 0.579 ppm), M18b (10.12 min, −1.132 ppm), M18c (13.70 min, −0.297 ppm) and M18d (13.93 min, −1.007 ppm) both showed [M−H]^−^ ion at *m*/*z* 479, which was 176 Da heavier than that of M19, indicating M18 was the glucuronide conjugation of M19. In the MS/MS spectrum, M18 produced three major fragment ions at *m*/*z* 303.0877, 289.0716 and 175.0249. The ion at *m*/*z* 303.0877 was generated through a neutral loss of 176 Da (GluA), the ion at *m*/*z* 175.0249 was the [M − H]^−^ ion of GluA, and the ion at *m*/*z* 289.0716 was the characteristic ion of C/EC.

#### 2.2.5. Sulfated Metabolites (M12)

M12a (7.90 min, 1.872 ppm), M12b (8.22 min, 2.279 ppm), M12c (13.89 min, −0.946 ppm) and M12d (14.68 min, −1.352 ppm) exhibited [M − H]^−^ ion at *m*/*z* 369, which were 80 Da heavier than that of the M17, suggesting M12 were the sulfate conjugations of C/EC. The major fragment ion at *m*/*z* 289.0723 was the characteristic ion of C/EC.

#### 2.2.6. Glucuronidated Metabolites (M10)

M10a (7.60 min, −0.586 ppm), M10b (8.12 min, 1.973 ppm), M10c (8.47 min, −0.715 ppm) and M10d (9.53 min, 0.790 ppm) gave the [M − H]^−^ ion at *m*/*z* 465, which was an increase of 176 Da (GluA) when compared with that of M17. In the MS/MS spectrum, M10 gave rise to the characteristic ion of C/EC at *m*/*z* 289.0719. Based on these data, M10 should be the glucuronidated product of C/EC.

#### 2.2.7. Oxidative Metabolites (M4, M9)

M4 (1.96 min, −0.315 ppm) displayed [M + H]^+^ ion at *m*/*z* 158.0811, which was 16 Da (O) heavier than that of M2. In the MS/MS spectrum, M4 gave rise to two main fragment ions at *m*/*z* 140.0529 [M + H-OH-H_2_]^+^ and *m*/*z* 112.0870 [M + H-COOH-H_2_]^+^. Therefore, M4 might be the oxide of M2.

M9 (5.79 min, −1.103 ppm) exhibited [M + H]^+^ ion at *m*/*z* 172.0966, which was 16 Da (O) higher than that of M6. In the MS/MS spectrum, M9 produced the fragment ions at *m*/*z* 154.0866 [M + H-OH-H_2_]^+^, 140.0710 [M + H-OCH_3_-H_2_]^+^ and 112.0759 [M + H-C_2_H_4_O_2_]^+^. Thus, M9 was tentatively identified as the oxide of M6.

#### 2.2.8. Mercapturic Acid-Conjugated Metabolites (M7)

M7 (4.31 min, −1.000 ppm) exhibited the [M + H]^+^ ion at *m*/*z* 319.1319. In the MS/MS spectrum, M7 produced four major fragment ions at *m*/*z* 287.1068 [M + H-CH_2_-H_2_O]^+^, 277.1223 [M + H-COCH_3_]^+^, 190.0901 [M + H-C_5_H_7_NO_3_]^+^ and 156.1022 [M + H-C_5_H_9_NO_3_S]^+^. The ion at *m*/*z* 156.1022 was the characteristic ion of arecoline. In accordance with [30], M8 was tentatively identified as arecoline mercapturic acid.

#### 2.2.9. Oxidative and Mercapturic Acid Conjugated Metabolites (M13)

M13 (8.33 min, −0.369 ppm) displayed the [M + H]^+^ ion at *m*/*z* 335.1270, which was 16 Da (O) heavier than M7. In the MS/MS spectrum, M13 gave rise to five major fragment ions at *m*/*z* 317.1172 [M + H-H_2_O]^+^, 303.1019 [M + H-H_2_O-CH_2_]^+^, 293.1173 [M + H-COCH_3_]^+^, 206.0851 [M + H-C_5_H_7_NO_3_]^+^ and 172.0973 [M + H-C_5_H_9_NO_3_S]^+^. Among the product ions, the ion at *m*/*z* 172.0973 was the typical ion of arecoline N-oxide (M10). Therefore, M13 was tentatively identified as arecoline N-oxide mercapturic acid.

#### 2.2.10. Dimer (M8)

M8 (5.58 min, −0.417 ppm) showed the molecular ion at *m*/*z* 343.1862, which was an increase of 171 Da compared to M9, suggesting M8 was the two dimer of M9. In the MS/MS spectrum, M8 produced the main fragment ion at *m*/*z* 172.0974, which was the characteristic ion of M9.

#### 2.2.11. Other Metabolites (M11, M14, M20)

M11 (7.67 min, −0.175 ppm) exhibited the [M + H]^+^ ion at *m*/*z* 144.1019, which was 2 Da heavier than that of arecaidine, indicating it was hydrogenated product of arecaidine. In the MS/MS spectrum, it produced three major fragment ions at *m*/*z* 126.0916 [M + H-OH]^+^, 98.0965 [M + H-COOH]^+^ and 84.0807 [M + H-CH_3_-COOH]^+^. Accordingly [30], M11 was proposed to be *N*-methylnipecotic acid.

M20 (13.06 min, −0.271 ppm) displayed the [M + H]^+^ ion at *m*/*z* 184.0968, and in the MS/MS spectrum it yielded the major fragment ions at *m*/*z* 166.0865 [M + H-H_2_O]^+^ and 152.0710 [M + H-H_2_O-CH_3_]^+^. It was tentatively identified as epinephrine (EP).

M14 (8.41 min, −0.057 ppm) showed the [M + H]^+^ ion at *m*/*z* 170.0812, which was a loss of 14 Da (CH_2_) compared with M20. In the MS/MS spectrum, M14 gave rise to the major fragment ion at *m*/*z* 152.0711 [M + H-H_2_O]^+^. Thus, M14 was tentatively identified as confirmed as norepinephrine.

## 3. Materials and Methods

### 3.1. Chemicals and Materials

*Areca catechu* L. nut was obtained from Tongrentang Pharmaceutical Co., Ltd. (Beijing, China), and authenticated by Professor Jingjuan Wang (Beijing University of Chinese Medicine). HPLC-grade acetonitrile, methanol and formic acid were purchased from Fisher Scientific. High pure water was obtained from Watson’s Food & Beverage (Guangzhou, China). All other reagents were of analytical grade and commercially available.

### 3.2. Preparation of the Areca catechu L. Nut Extract

*Areca catechu* L. nut was ground into a crude powder. At room temperature, 100 g powdered nut was soaked in 1 L water for 1 h. After filtering, the residue was added to 0.8 L water for further extraction under the same conditions. The two filtrates were combined and evaporated to the final volume of 20 mL in a vacuum at 45 °C to obtain an oral solution (5 g/mL). For LC/MS analysis, the *Areca catechu* L. nut extract (5 g/mL) was diluted to a concentration of 10 mg/mL with water, and then the solution was filtered through a 0.22 μm membrane (pore size).

### 3.3. Animal and Sample Collection

Sprague-Dawley rats (male, 200–250 g) were provided by Beijing Vital River Laboratory Animal Technology Co., Ltd. (Beijing, China). All animals were kept in an environmentally controlled breeding room at 23 °C, 60 ± 5% humidity with room-lights alternated on a 12-h dark–light cycle. Water and standard diet were provided ad libitum. The animal facilities and protocols were approved by the Institutional Animal Care and Use Committee of Beijing University of Chinese Medicine.

Rats were randomly separated into two groups of three animals each and were acclimated in metabolic cages for three days. Prior to the experiment, the rats were fasted overnight with unlimited access to water. Then, the treatment group rats were given ACNE intragastrically at a single dose of 50 g/kg (crude drug weight/rat weight). Blank control group rats were orally administered with equal dose of saline at the same time. The animals were continuously administered twice per day. Then, 1.5 h after the seventh drug administration, the animals were anesthetized with chloral hydrate (400 mg/kg) by intraperitoneal injection. Then, blood samples were collected from the abdominal aorta. Urine samples were collected every 12 h after the first drug administration, then, all urine samples from each animal were combined into one sample. After the experiments, all rats were sacrificed by performing a bilateral thoracotomy.

### 3.4. Sample Preparation

Each blood sample was centrifuged (5000 rpm, 4 °C ) for 10 min to obtain the plasma, and then treated by addition of methanol in the ratio of 1:3 (*v*/*v*) to precipitate protein. The mixture was performed by vortexing for 10 min and centrifuging (10,000 rpm, 4 °C) for 15 min. The supernatant was transferred to another clean tube, and dried under a gentle flow of nitrogen at 40 °C. Then the dried residue was reconstituted with 200 μL of methanol for UHPLC-LTQ-Orbitrap analysis.

Each urine sample (10 mL) was concentrated to the final volume of 5 mL in a vacuum. Then, 500 microL urine were mixed with 1.5 mL of methanol and sonicated in an ultrasonic bath for 0.5 h, and then centrifuged (10,000 rpm, 4 °C) for 10 min. The supernatant was transferred to another clean tube, and dried under a gentle flow of nitrogen at 40 °C. The dried residue was reconstituted with 200 μL of methanol for UHPLC-LTQ-Orbitrap analysis.

### 3.5. Instrumentation and Analytical Conditions

Sample analyses were performed using an ultimate 3000 LC system coupled to an LTQ Orbitrapmass spectrometer via an electrospray ionization (ESI) interface. The chromatography system consisted of an autosampler, a diode array detector, a column compartment and two pumps. Xcalibur, Metworks and Mass Frontier 7.0 software packages (Thermo Corporation, Waltham, MA, USA) were employed for data collection and data analysis.

Chromatographic separations were performed on a Thermo Scientific BOS Hypersil C_18_ column 2.1 × 150 mm^2^, with 2.4-μm particle size. The mobile phases A and B comprised 0.1% formic acid in water and acetonitrile, respectively. The LC gradient program (time (in min) %mobile phase B) was set at 0.01/2, 5/2, 5.1/10, 10/10, 25/80, 26/2, and 31/2. The chromatographic runs were performed at a flow rate of 0.3 mL/min. The injection volume was 3 μL with column oven at 35 °C.

The ESI source parameters were as follows: the capillary temperature was 250 °C, source voltage and the spray voltage were set at 5 kV, and sheath gas (N_2_) flow was 35 psi. The ESI source was operated in both negative and positive ionization mode. In the Fourier transform (FT) cell, full MS scans were acquired in the range of *m*/*z* 50–2000. The MS/MS experiments were set as data-dependent scans.

## 4. Conclusions

In this study, a sensitive LC/MS method was established for the comprehensive analysis and profiling of the multiple components of ACNE and their absorption and metabolite components in rats after oral administration. In total, 12 components were detected and identified on the basis of the retention times on the UHPLC system, the accurate molecular mass, and characteristic product ions. After oral administration of ACNE to rats, a total of 40 exogenous chemical compounds were identified in urine and plasma, including 8 prototypes and 32 metabolites. The major metabolic reactions of ACNE were proposed, including methylation, sulfation, glucuronidation, oxidation, and mercapturic acid conjugation. All above mentioned results laid a solid foundation for further pharmacological and toxicological research of *Areca catechu* L. nut, which will facilitate its clinical usage and drug discovery.

## Figures and Tables

**Table 1:** The mass spectral data of components observed by UHPLC-LTQ-Orbitrap.

Component	*t*_R_ (Min)	[M + H]^+^ Ion (*m*/*z*)	Error (ppm)	Formula	Fragment Ions (*m*/*z*)	Identification
**1**	1.36	118.0861	−1.568	C_5_H_11_NO_2_	72.0806	Valine
**2**	1.70	128.0705	−0.743	C_6_H_9_NO_2_	110.0601, 81.0334	Guvacine/Isoguvacine
**3**	1.74	142.0862	−0.085	C_7_H_11_NO_2_	128.0745, 124.0758, 96.0807, 81.0333	Arecaidine
**4**	1.87	144.1018	−0.869	C_7_H_13_NO_2_	126.0915, 114.0913, 84.0806	Methyl piperidine-3-carboxylate
**5**	3.05	182.0810	−0.822	C_9_H_11_NO_3_	165.0548, 136.0760	Tyrosine
**6**	3.47	142.0862	−0.115	C_7_H_11_NO_2_	113.9638, 110.0602, 81.0334	Guvacoline
**7**	4.09	156.1018	−0.115	C_8_H_13_NO_2_	124.0757, 113.0597, 96.0807, 81.0333	Arecoline
**8**	7.81	156.1017	−0.255	C_8_H_13_NO_2_	128.1069, 113.0597, 81.0333	Arecolidine
**9**	8.68	579.1489	−1.334	C_30_H_26_O_12_	453.1197, 427.1039, 409.0937, 291.0874	Procyanidin B2
**10**	9.48	291.0861	−0.669	C_15_H_14_O_6_	273.0762, 165.0548, 151.0392, 139.0392, 123.0443	Catechin (C)
**11**	9.53	205.0970	−0.996	C_11_H_12_N_2_O_2_	188.0917, 159.9331	Tryptophan
**12**	12.20	291.0860	−0.978	C_15_H_14_O_6_	273.0764, 165.0549, 151.0393, 139.0393, 123.0444	Epicatechin (EC)

*t*_R_: retention time.

**Table 2:** The mass spectral data of metabolites observed by UHPLC-LTQ-Orbitrap.

Metabolite	*t*_R_ (Min)	Selected Ion	Calculated Mass (*m*/*z*)	Error (ppm)	Formula	Fragmentation	Identification	Source
M1	1.75	[M + H]^+^	128.0706	−0.352	C_6_H_9_NO_2_	110.0601, 81.0332	Guvacine/Isoguvacine	Urine; Plasma
M2	1.76	[M + H]^+^	142.0862	−0.740	C_7_H_11_NO_2_	124.0759, 96.0809, 81.0334	Arecaidine	Urine; Plasma
M3	1.78	[M + H]^+^	144.1018	−0.661	C_7_H_13_NO_2_	126.0915, 114.0915, 84.0806	Methyl piperidine-3-carboxylate	Urine; Plasma
M4	1.96	[M + H]^+^	158.0811	−0.315	C_7_H_11_NO_3_	140.0529, 112.0870	Arecaidine N-oxide	Urine; Plasma
M5	3.28	[M + H]^+^	142.0862	−0.388	C_7_H_11_NO_2_	113.0597, 110.0601, 81.0333	Guvacoline	Urine; Plasma
M6	3.58	[M + H]^+^	156.1018	−0.418	C_8_H_13_NO_2_	124.0758, 113.0598, 96.0807, 81.0333	Arecoline	Urine
M7	4.31	[M + H]^+^	319.1319	−1.000	C_13_H_22_N_2_O_5_S	287.1068, 277.1223, 190.0901, 156.1022	Arecoline mercapturic acid	Urine; Plasma
M8	5.58	[M + H]^+^	343.1862	−0.417	C_16_H_26_N_2_O_6_	172.0974	Arecoline N-oxide dimer	Urine; Plasma
M9	5.79	[M + H]^+^	172.0966	−1.103	C_8_H_13_NO_3_	154.0866, 140.0710, 112.0759	Arecoline N-oxide	Urine; Plasma
M10a	7.60	[M − H]^−^	465.1025	−0.586	C_21_H_22_O_12_	289.0719	Glucuronidation of C/EC	Urine
M11	7.67	[M + H]^+^	144.1019	−0.175	C_7_H_13_NO_2_	126.0916, 98.0965, 84.0807	N-methylnipecotic acid	Urine; Plasma
M12a	7.90	[M − H]^−^	369.0282	1.872	C_15_H_14_O_9_S	289.0723	Sulfation of C/EC	Urine
M10b	8.12	[M − H]^−^	465.1037	1.973	C_21_H_22_O_12_	289.0721	Glucuronidation of C/EC	Urine
M12b	8.22	[M − H]^−^	369.0282	2.279	C_15_H_14_O_9_S	289.0723	Sulfation of C/EC	Urine
M13	8.33	[M + H]^+^	335.1270	−0.369	C_13_H_22_N_2_O_6_S	317.1172, 303.1019, 293.1173, 206.0851, 172.0973	Arecoline N-oxide mercapturic acid	Urine; Plasma
M14	8.41	[M + H]^+^	170.0812	−0.057	C_8_H_11_NO_3_	152.0711	Norepinephrine	Urine; Plasma
M10c	8.47	[M − H]^−^	465.1024	−0.715	C_21_H_22_O_12_	289.0716	Glucuronidation of C/EC	Plasma
M15	8.60	[M + H]^+^	579.1491	−1.023	C_30_H_26_O_12_	453.1199, 427.1041, 409.0938, 291.0875	Procyanidin B2	Urine; Plasma
M16a	9.20	[M − H]^−^	383.0435	1.020	C_16_H_16_O_9_S	303.0879, 137.0249	Sulfation + methylation of C/EC	Urine
M17a	9.31	[M + H]^+^	291.0865	0.602	C_15_H_14_O_6_	273.0766, 165.0550, 151.0394, 139.0394, 123.0444	Catechin(C)	Urine; Plasma
M10d	9.53	[M − H]^−^	465.1031	0.790	C_21_H_22_O_12_	289.0721	Glucuronidation of C/EC	Plasma
M18a	9.86	[M − H]^−^	479.1187	0.579	C_22_H_24_O_12_	303.0877, 289.0716, 175.0249	Methylation + glucuronidation of C/EC	Urine
M18b	10.12	[M − H]^−^	479.1179	−1.132	C_22_H_24_O_12_	303.0873, 289.0715, 175.0247	Methylation + glucuronidation of C/EC	Urine
M19a	10.16	[M + H]^+^	305.1020	0.083	C_16_H_16_O_6_	287.0921, 179.0707	Methylation of C/EC	Urine; Plasma
M19b	10.50	[M + H]^+^	305.1021	0.280	C_16_H_16_O_6_	287.0923, 179.0708	Methylation of C/EC	Urine; Plasma
M16b	10.73	[M − H]^−^	383.0430	−0.259	C_16_H_16_O_9_S	303.0876, 137.0249	Sulfation + methylation of C/EC	Urine
M16c	11.73	[M − H]^−^	383.0427	−1.225	C_16_H_16_O_9_S	303.0882, 137.0250	Sulfation + methylation of C/EC	Urine
M17b	11.99	[M + H]^+^	291.0864	0.293	C_15_H_14_O_6_	273.0767, 165.0551, 151.0394, 139.0394, 123.0445	Epicatechin(EC)	Urine; Plasma
M16d	12.72	[M − H]^−^	383.0429	−0.729	C_16_H_16_O_9_S	303.0882, 137.0250	Sulfation + methylation of C/EC	Urine
M19c	12.94	[M + H]^+^	305.1020	−0.015	C_16_H_16_O_6_	287.0923, 179.0708	Methylation of C/EC	Urine; Plasma
M16e	13.01	[M − H]^−^	383.0436	1.334	C_16_H_16_O_9_S	303.0876, 137.0250	Sulfation + methylation of C/EC	Plasma
M20	13.06	[M + H]^+^	184.0968	−0.271	C_9_H_13_NO_3_	166.0865, 152.0710	Epinephrine(EP)	Urine
M16f	13.29	[M − H]^−^	383.0432	0.289	C_16_H_16_O_9_S	303.0875, 137.0249	Sulfation + methylation of C/EC	Plasma
M18c	13.70	[M − H]^−^	479.1183	−0.297	C_22_H_24_O_12_	303.0876, 289.0718, 175.0249	Methylation + glucuronidation of C/EC	Plasma
M12c	13.89	[M − H]^−^	369.0271	−0.946	C_15_H_14_O_9_S	289.0722	Sulfation of C/EC	Plasma
M18d	13.93	[M − H]^−^	479.1179	−1.007	C_22_H_24_O_12_	303.0876, 289.0717, 175.0249	Methylation + glucuronidation of C/EC	Plasma
M21	14.27	[M + H]^+^	321.0943	−2.549	C_16_H_16_O_7_	303.1331, 289.0689	Methylation of epigallocatechin(EGC)	Urine
M16g	14.34	[M − H]^−^	383.0432	2.378	C_16_H_16_O_9_S	303.0883, 137.0250	Sulfation + methylation of C/EC	Plasma
M19d	14.35	[M + H]^+^	305.1018	−0.507	C_16_H_16_O_6_	287.0920, 179.0706	Methylation of C/EC	Urine
M12d	14.68	[M − H]^−^	369.0270	−1.352	C_15_H_14_O_9_S	289.0722	Sulfation of C/EC	Plasma

## Supplementary Materials

Supplementary materials are available online. Figure S1: Extracted ion chromatogram of each major component identified in ACNE.
