# Supplementary figures and images for: Screening and Identification of the Metabolites in Rat Plasma and Urine after Oral Administration of Areca catechu L. Nut Extract by Ultra-High-Pressure Liquid Chromatography Coupled with Linear Ion Trap–Orbitrap Tandem Mass Spectrometry
Source: Molecules. 2017 Jun 21;22(6):1026. doi: 10.3390/molecules22061026 (PMC6152711; doi:10.3390/molecules22061026)

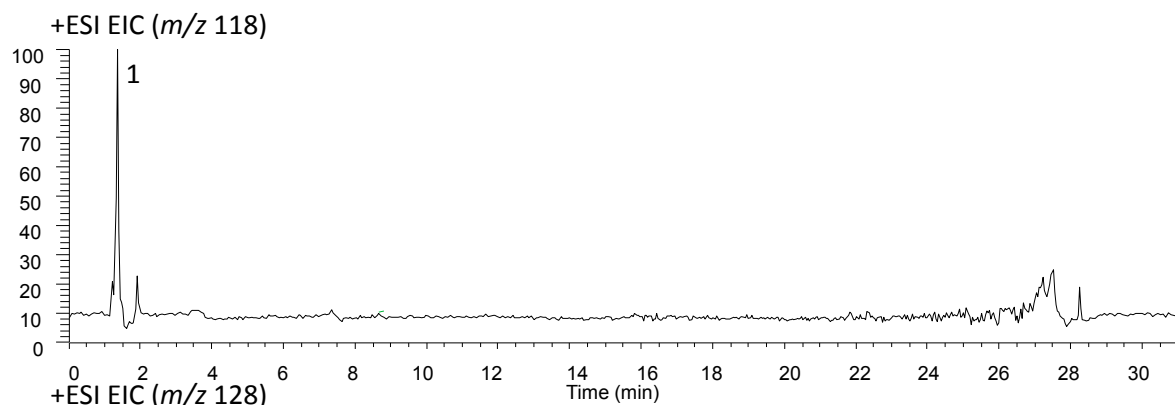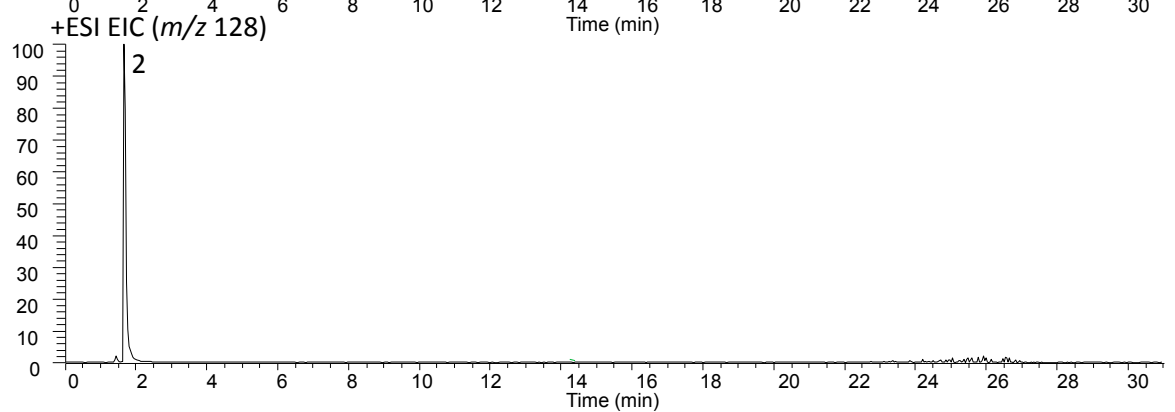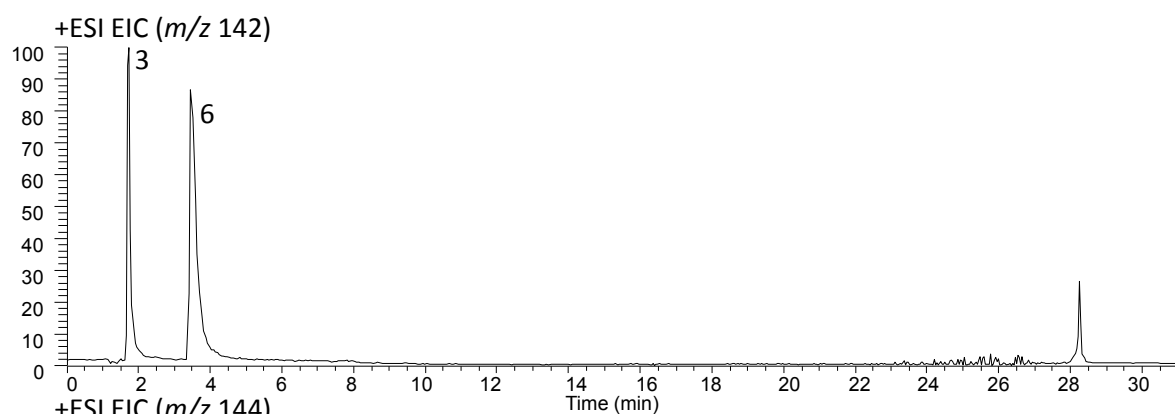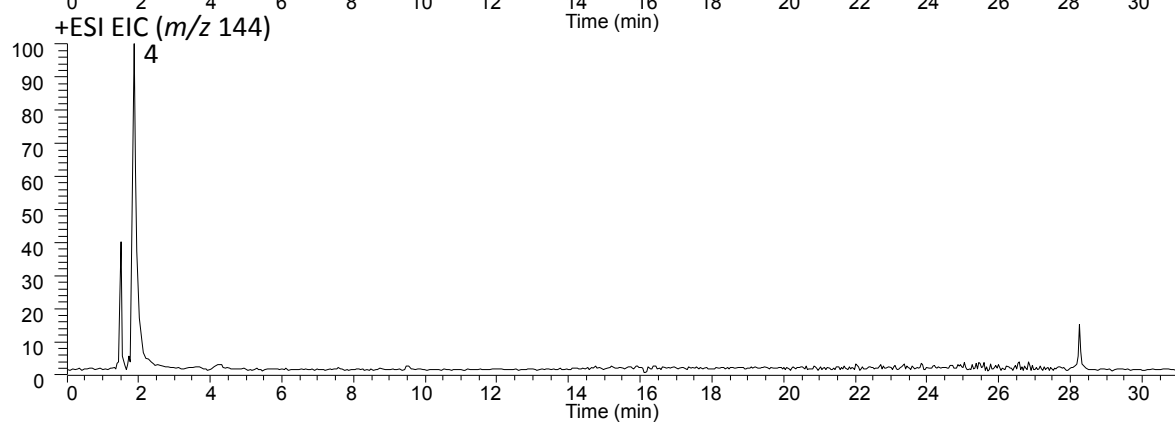

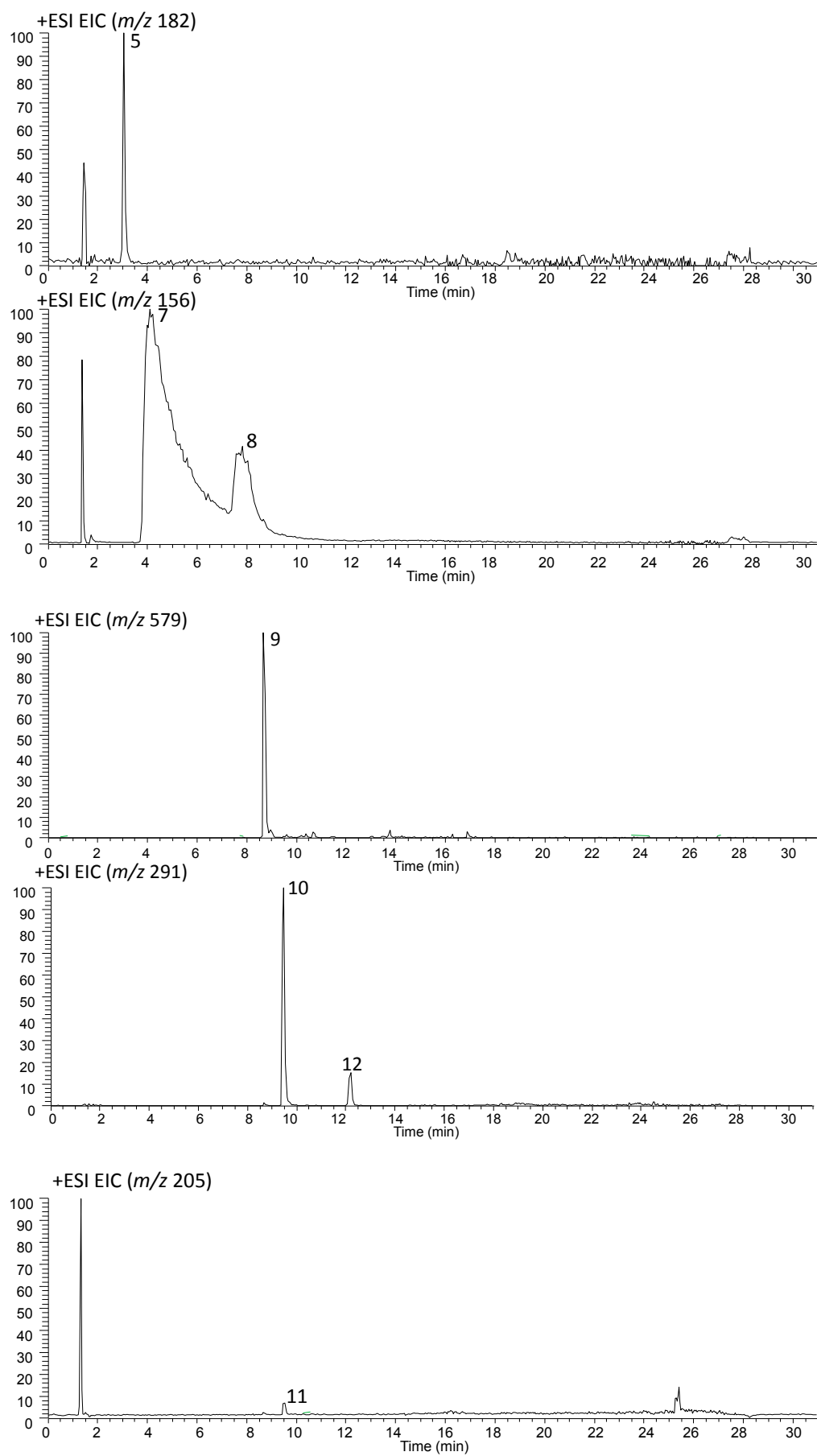

**Figure S1.** Extracted ion chromatogram of each major component identified in ACNE.

Supplement: Supplementary file 1 [file molecules-22-01026-s001.pdf]
